# Dental approach of orofacial pain in head and neck cancer patients

**DOI:** 10.4317/jced.55311

**Published:** 2018-11-01

**Authors:** Diele Arantes, Náira Costa, Tacyana Resende, Karina Mikulas, Pierre da Silva Júnior, Rafael Brito, Vladimir Noronha, Roberto Pedras, Luciana Corrêa

**Affiliations:** 1 DDS, PhD. School of Dentistry, Centro Universitário Newton Paiva, Av. Silva Lobo, 1718, Nova Granada, Belo Horizonte, MG 30431-262, Brazil; 2 DDS. School of Dentistry, Centro Universitário Newton Paiva, Av. Silva Lobo, 1718, Nova Granada, Belo Horizonte, MG 30431-262, Brazil; 3 School of Dentistry, Centro Universitário Newton Paiva, Av. Silva Lobo, 1718, Nova Granada, Belo Horizonte, MG 30431-262, Brazil; 4 DDS, MSc. School of Dentistry, Centro Universitário Newton Paiva, Av. Silva Lobo, 1718, Nova Granada, Belo Horizonte, MG 30431-262, Brazil; 5 DDS, PhD. School of Dentistry, University of São Paulo. Av. Prof. Lineu Prestes, 2227, Cidade Universitária, São Paulo, SP 05508-000, Brazil

## Abstract

**Background:**

Orofacial pain (OFP) is an undesirable sensation frequently associated with head and neck cancer (HNC) and its treatment. OFP negatively impacts the quality of life of oncological patients. The approach to OFP diagnosis and management can differ if the patient visits a dentist or physician. The aim of this study was to present a case series of HNC with OFP managed by a dentist team and to discuss its role in the management of OFP.

**Material and Methods:**

We recruited twenty-two adult patients with OFP and previous diagnosis of HNC referred to an academic dental clinic from 2015 to 2017.

**Results:**

Nociceptive was more frequent than mixed and neuropathic pain, however 54,4% of the cases showed a neurological component. All types of pain were managed by dentist through removal of pain’s cause and appropriated local and systemic treatment. The intensity of pain was reduced in 86,3% of patients, and 45,4% of them reported absence of pain at the end of treatment.

**Conclusions:**

Dentist’s assessment plays a distinct and crucial role in the diagnosis and management of OFP in HNC patients throughout the oncological treatment.

** Key words:**Pain, orofacial, neoplasm, head and neck, dentist.

## Introduction

Head and neck cancer (HNC) are a group of malignant neoplasms that arises in the oral cavity (40%), oropharynx and hypopharynx (15%), and larynx (25%) (1). Orofacial pain (OFP) is reported by approximately half of HNC patients prior to oncology therapy, 81% during treatment, 70% at the end of therapy and 36% six months after treatment (2). Ideally physicians and dentists should work in collaboration to obtain a precise diagnosis and determine the best approach to OFP. The management plan may be different depending on which specialist the patients seeks for consultation, because the pain could involve dental diseases, neurological problems, co-morbidities, depression and other forms of chronic pain. Moreover, a biopsychosocial approach is advised to achieve a treatment (3).

According to the National Institute of Health (2016) “pain is an undesirable sensation of discomfort, an unpleasant human experience that decreases the quality of life in patients with cancer” (4). Therefore, pain negatively affects cancer patients and their relatives (5). Cancer-induced pain may be due to nociceptive, neuropathic or mixed mechanisms. Nociceptive pain is related to involvement of specific structures like bone, muscle or viscera. Neuropathic pain is due to involvement of peripheral or central afferent neural pathways. Mixed mechanisms present more than one element of the nociceptive and neuropathic features (6).

OFP may be caused by direct cancer involvement of anatomical structures, toxicity of oncological treatment or patient comorbidities (2,3). In the oral cavity, overlooked dental and periodontal disorders prior to cancer therapy are the commonest acute causes of OFP in HNC patients (7). Oral mucositis and stomatitis are also a frequent acute adverse effect of combined chemotherapy and radiotherapy (CRT). In this context, the incidence of severe mucositis ranges between 60% and 90%, which significantly increases pain during anti-cancer treatment (8). Temporomandibular disorders (TMD) cause musculoskeletal pain and frequently affect HNC patient. Trismus is a TMD resulted from damage of the masticatory muscles, a late complication of head and neck radiotherapy (9). Burning mouth syndrome (BMS) and painful post-traumatic trigeminal neuropathy, chronic neuropathic conditions characterized by unilateral or bilateral facial or burning oral pain of the tongue and other parts of the oral mucosa, could be secondary to nerve damage induced by CRT (10). Detection and proper management of those oral disorders requires a dental specialist with experience in the management of oncological patients.

Preventive measures and effective analgesia are the basis of multidisciplinary cancer pain management (2,3). The ability to provide an efficient treatment depends on meticulous clinical exam, knowledge of pharmacological and non-pharmacological agents and recognition of pain episodes (7).

This study aimed to report our experience in the diagnosis and management of OFP in HNC patients referred to an academic dental outpatient clinic and to discuss the importance of a dentist in the multidisciplinary approach to oncological patients with OFP.

## Material and Methods

This study was approved by the Institutional Ethics and Research Committee (protocol number 904.529). We recruited adult patients with OFP and previous diagnosis of HNC referred to an academic dental clinic from March 2015 to November 2017. Eligible patients that accepted to participate and provided an informed consent entered the study.

-Dentist’s structured interview 

Clinical examination started with a structured interview to assess demographic data; type, localization and stage of HNC; timing of oral assistance (before, during, or after oncological treatment); modality (surgery, chemotherapy, and radiotherapy) and duration of oncological therapy; smoking and alcohol abuse; and co-morbidities.

The OFP history included the following data: location and radiation (dental, mouth mucosa, musculoskeletal, and neurological); timing (onset, duration, and periodicity); quality (LANSS - Leeds assessment of neuropathic symptoms and signs – scale part A) (11); intensity (VAS - visual analog scale) (12); modifying factors of pain (relieving and aggravating factors); and previous attempts of pain management.

Patients with HNC rated pain intensity, according to a validated VAS (12). The use of 0 to 100 mm VAS scale was explained for patient self-assessment (12,13). The wording used for pain intensity assessment was “no pain” and “worst pain imaginable”. We described their pain intensity based in cut-off points on VAS: no pain (0–4 mm), mild pain (5–44 mm), moderate pain (45–74 mm), and severe pain (75–100 mm) (14). Pain intensity was measured in each consult and recorded for comparative analysis in the first (VAS1) and last dental query (VAS2).

-Oral and maxillofacial examination

A dentist with expertise in orofacial pain realized a complete face, head, neck, and oral cavity examination by inspection, palpation, percussion, and auscultation. Mandibular active range of motion, standardized temporomandibular joint, masticatory and cervical muscle palpation were also done, according to research diagnostic criteria for temporomandibular disorders (RDC/DTM) (15). For nociceptive or neuropathic pain differentiation LANSS scale part B was used (11).

Dental and periodontal examination was done by a combination of clinical and radiographic analysis. Cold stimuli to the clinical crown by Endo Ice spray was done when caries, restorations (defective, newly placed) or tooth pain history was present. Periodontal status was defined after probing and dental mobility analysis.

-Dentist diagnosis and management

The types of dental treatment protocols were categorized according to anti-cancer therapy timing (16-18). For patients with dental pain diagnosed before oncological treatment and without time constraint or systemic limitations, a complete protocol was indicated. This protocol included: 1. Repair of all teeth with caries; 2. Extraction of teeth with severe caries, apical periodontitis, advanced periodontal disease (probing depth ≥ 6 mm and/or furcation I, II, III), mobile primary teeth (>50% root resorption), and partially erupted third molars. Partial protocol was the best option for patients with time constraint or systemic limitations and dental pain diagnosed before CRT. This protocol differed from complete protocol in that: 1. mild/ moderate caries were restored depending on time availability; 2. Extraction of teeth with severe caries, apical periodontitis (symptomatic lesion and lesions ≥ 5 mm), advanced periodontal disease (probing depth ≥ 8 mm, mobility III, severe inflammation), severe mobility, and partially erupted third molars with purulent secretions of pericoronitis. Intervention only in dental pain was the option for patients that did not have time or systemic conditions to complete or partial dental treatment and for patients during CRT. For patients who manifest severe dental pain or give a previous history of pain in teeth with symptoms of mild or moderate pain and had not systemic problems, extraction was indicated. Another dental pathology, if present, was monitored during anti-neoplastic therapy.

The complete dental treatment protocol was also the option for patients after CRT. For patients with indication of tooth extraction in the field of radiation were prescribed the osteonecrosis prevention protocol which included amoxicillin (2 g) or clindamycin (600 mg) taken orally one hour before the surgery and half of dose every eight hours for seven days after extraction; and antibacterial mouthwashes with 10 ml of chlorhexidine gluconate 0.12% solution for one minute twice a day starting seven days before extraction and continued for seven days after extraction (17,18).

Severity of oral mucositis was defined by World Health Organization (WHO) (8) as Grade 0: none on oral mucosa; Grade 1: erythema and soreness, no ulcer; Grade 2: oral erythema, ulcers, solid diet tolerated; Grade 3: oral ulcers, liquid diet only; Grade 4: unable to tolerate a solid or liquid diet. Among the few mucositis prevention and treatment measures available, photobiomodulation (PBM) with low-level laser therapy (LLLT) was applied (19). The PBM protocol for treatment of oral mucositis was 660 and 808 nm, 100mW, 2J/cm2 on the lesions, once a week.

TMD was managed through thermotherapy (10 minutes of warm water compression at the pain site, three times a day) (20), oral muscle relaxant agents and adjuvants, and systemic analgesics as needed according to WHO analgesic ladder (21,22).

Diagnostic anesthetic blocks (23) were useful in localizing symptoms in some cases of neurophatic pain. Pharmacological management of neurophatic pain followed WHO analgesic ladder (21). Anticonvulsants (carbamazepine, gabapentin), antidepressants, and other adjunctive medications were also added on an individual basis (3).

## Results

-Case series

Twenty-two patients (eighteen male/four female) agreed to participate in the study ( Table 1,  Table 1 continue,  Table 1 continue-1). Mean age was fifty years (range: eighteen to seventy-three years-old). Twenty-one patients had SCC (squamous cell carcinoma) and one patient had cervical Hodgkin’s lymphoma. The primary neoplasm was in advanced stage and their locations were: oropharynx (22,7%); tongue (22,7%); larynx (13,6%), maxillary sinus (9%); amygdala (9%); parapharynx space (4,5%); mandible (4,5%); cheek (4,5%); mouth floor (4,5%) and cervical lymph node (4,5%). Thirteen patients (59%) had undergone cancer therapy previously. Four patients (18,1%) were still undergoing oncological treatment at the first dental consultation. Five patients (22,7%) had not started cancer therapy before referral to our dental clinic. Eighteen patients (81,8%) received CRT during one to eight months, and ten (45,4%) also underwent surgical resection.

**Table 1:**
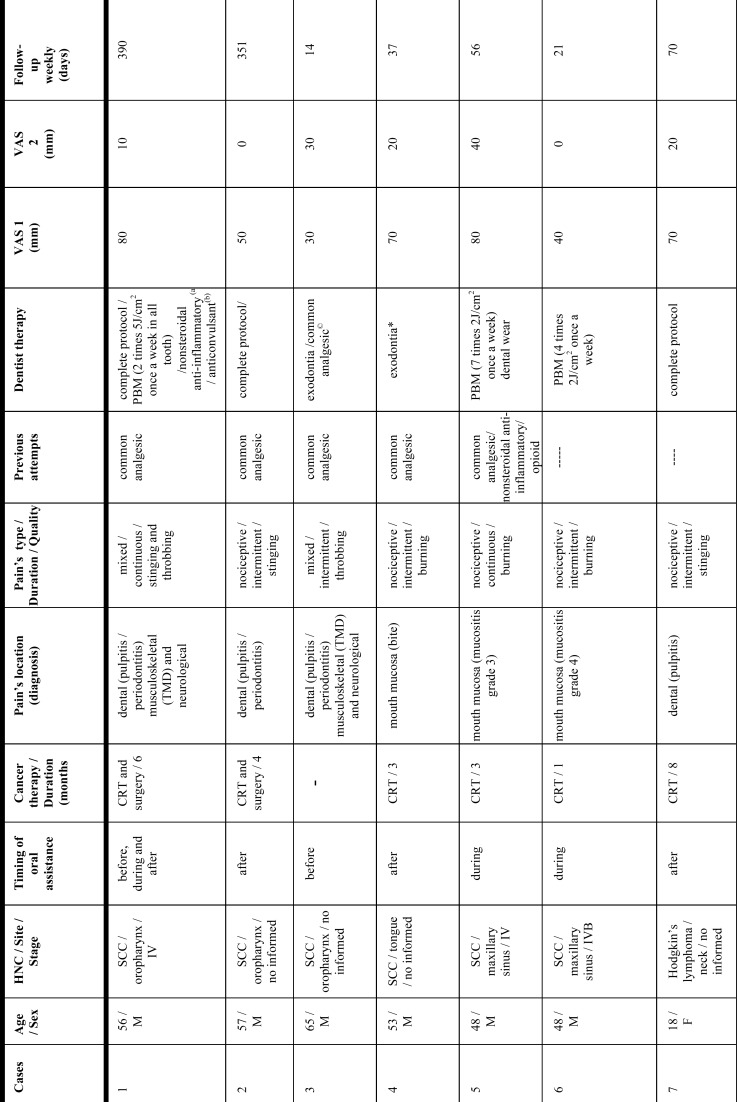
Characteristics of case series of the current study.

**Table 1 continue:**
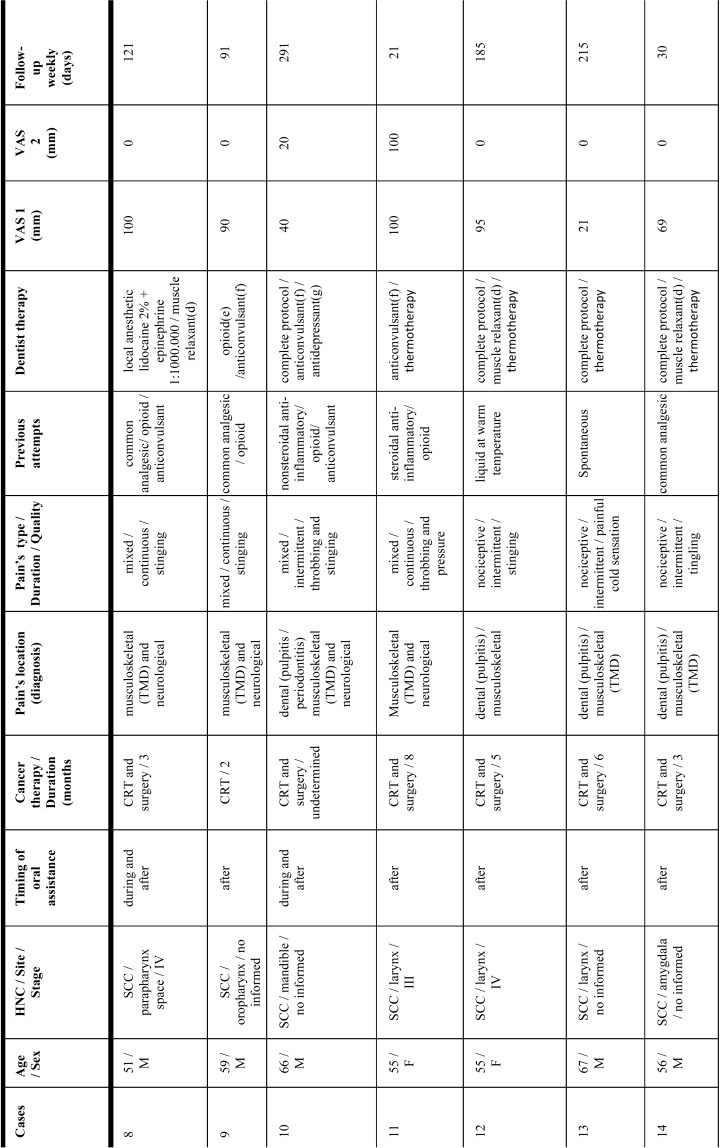
Characteristics of case series of the current study.

**Table 1 continue-1:**
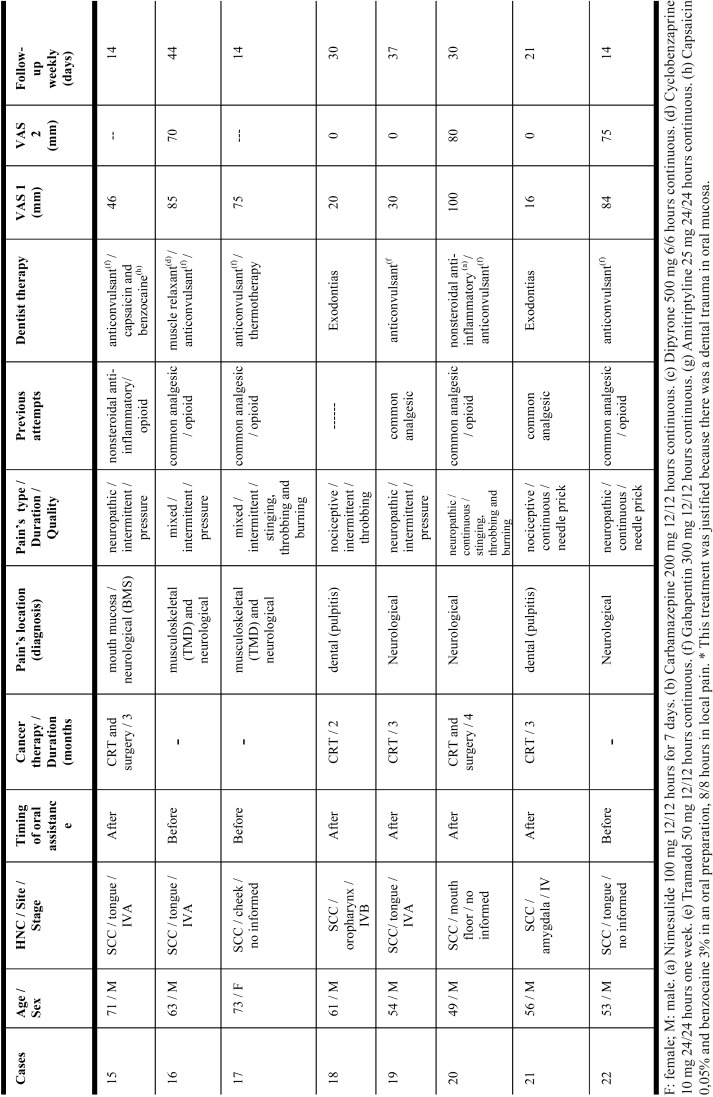
Characteristics of case series of the current study.

The majority of patients reported alcohol (86,3%) and tobacco (90,9%) consumption. Ten patients (45,4%) interrupted this consumption after malignancy diagnosis. Nine patients (40,9%) presented co-morbidities: arterial hypertension (18,1%), urinary tract infection (4,5%), hypothyroidism (4,5%), diabetes mellitus type II (4,5%), depression (4,5%), gallbladder stones (4,5%) and gastritis (4,5%).

-Pain diagnosis and management

OFP was associated with more than one diagnosis in 50% of the patients. The etiologies included: neurological (54,5%), musculoskeletal (50%), pulpitis (45,4%), periodontitis (18,1%), mucositis (9%) and bite in oral mucosa (4,5%). The most frequent type of pain was nociceptive (45,4%), followed by mixed type (36,3%) and neuropathic pain (18,1%). Eight patients (36,3%) reported continuous pain. In all other patients, pain was intermittent (63,6%) though lasting for more than two hours. Ten patients (45,5%) reported severe pain according to VAS. Five patients (22,7%) referred moderate pain and seven (31,8%) reported mild pain. Twenty patients (90,9%) reported a specific type of pain sensation: stinging (36,3%), throbbing (27,2%), pressure (18,1%), burning (18,1%), needle prick (9%), tingling (4,5%) and painful cold sensation (4,5%). Four patients (18,1%) complained simultaneously by two or more pain sensations.

Two patients (9%) reported spontaneous pain. The others (90,9%) had at least one pain trigger or aggravating factors: chew (59%) of fibrous food (36,3%); ice (50%), hot (36,3%), seasoned (22,7%) or sweet (22,7) food intake; open mouth movement (22,7%); stress and anxiety (18,1%); touch (13,6%); dental overload (13,6%); toothbrush (4,5%); decubitus (4,5%) and mouth breath (4,5%). Eighteen patients (81,8%) reported pain relief measures. Drugs were the most cited, including: standard oral analgesic (63,6%), opioids (45,4%), nonsteroidal anti-inflammatory drugs (13,6%), anticonvulsants (9%), oral corticoids (4,5%) and warm liquids (4,5%). Ten patients (45,4%) used a combination of two or more drugs.

OFP in 50% of the patients arose in dental structures due to pulpitis and periodontitis. In this group, exodontia was indicated in ten patients (45,4%), and one patient also underwent PBM (5J/cm2) once a week in all teeth because of dental sensibility. One patient was managed with fluoride therapy for white spot lesion in all teeth with sensibility and direct restoration.

Two patients presented tongue and labial mucositis and were treated with PBM (2J/cm2) once a week. In one patient, dental trauma in oral mucosa was diagnosed as the pain’s cause, and exodontia was undertaken. One patient presented BMS that was managed with topical capsaicin and benzocaine three times a day. Thermotherapy in pain site was recommended for five patients (22,7%).

Systemic pain medication was recommended to fourteen patients (63,6%), such as anticonvulsant (45,4%), muscle relaxant (18,1%), nonsteroidal anti-inflammatory (9%), common analgesic (4,5%), opioid (4,5%) and antidepressant (4,5%). For two patients (9%) a combination of two systemic pain drugs was recommended. One patient received intramuscular anesthetic (lidocaine 2% + epinephrine 1:100.000) to define the type of pain.

The follow-up ranged from fourteen to 390 days (mean 95,3 days). Ten patients (45,4%) reported absence of pain and six (27,2%) referred mild pain after oral care. Three patients (13,6%) maintained severe pain, although slightly reduced. One patient reported no response and two patients abandoned treatment.

## Discussion

The present case series reflects the typical scenario of HNC in Brazil, where oral cancer represents the fifth commonest malignant neoplasm in men. Tobacco smoking and alcohol consumption are the most prevalent risk factors, and its diagnostic still remains late in the majority of cases (24). In this context, the dentist’s role is important to reduce the acute and long-term side effects of CRT and surgery for HNC patients. OFP (4), trismus (9,22), osteoradionecrosis (16), oral infections (25), dysgeusia (9), dental demineralization and caries (16,26), mucositis (8), salivary gland hypofunction and xerostomia (22) are the most common oral issues that affect oncological patient’s quality of life. Poor dental health observed in HNC patients is the highest risk factor for many of these complications and should be prevented with dental prophylactic care and extraction of compromised teeth ahead of CRT and surgery (16,27). However, the great majority of patients still initiate cancer therapy without dental care assessment, a reality clearly demonstrated in the present study, where 45,4% of our patients presented pain associated with pulpitis and only five patients (22,7%) began the OFP management and dental treatment before oncological therapy.

OFP diagnosis is a challenging condition for both dentist and physician. General practitioners commonly refer OFP patients to ear, nose and throat, neurology, or pain medicine (3), whereas the diagnosis of dental and non-dental pain was the basic principle of our proposal.

According to Epstein *et al.*, clinical presentation of OFP depends on cancer stages and its associated therapy. At the beginning, pain could be associated with neoplasm and its growth or paraneoplastic neuropathy. Perineural spread of HNC can give trigeminal neuropathic symptomatology in up to 80% of patients (28). This occurs due to inflammation within closed spaces that may carry out nerve damage secondary to tumor pressure-induced ischemia (16). All patients in this study that reported pain before cancer therapy were diagnosed with neuropathic component, a feature that reinforces this hypothesis.

Pain during therapy may be associated with neural and mucosal damage due to cytotoxicity of CRT or post-surgery anatomical abnormalities (2,27). In this study, mucositis was present in two patients that started oral treatment during cancer therapy, leading to dysphagia and limitation to oral medications intake. Post-surgery pain was observed in seven patients (31,8%) with general status compromise, and with speech impairment due to pain and trismus. Post-surgical pain causes fatigue, depression, and impairs quality of life (2).

The majority of patients in the present study were in post-therapy period that is characterized by long-term consequences of cancer treatment (2,16). In this group we observed radiation caries that is commonly described when patients do not receive educative orientations and preventive oral care before cancer therapy (16). One patient of this study complained pain when opening the mouth due to sensibility in white spot lesion in cervical region of all teeth.

Definition of OFP’s type based on pathophysiological classification is essential to determine its appropriated pain management (28). Intermittent nociceptive somatic pain was the most frequent type of pain observed in this study, with a variety of sensations. In these cases, the pain’s localization was precise (tooth, oral mucosa, and musculoskeletal nerve complex) and pain trigger or aggravating factors were obvious. Nociceptive pain’s control was achieved by complete dental therapy or elimination of local pain. The results could be demonstrated in this study by a decrease in pain score observed at VAS2 in all patients with nociceptive pain.

In our series, a mixed-type OFP was diagnosed in some cases, with both neuropathic and nociceptive components resulting in variable pain characteristics. This pain was efficiently treated with analgesic and adjuvants drugs, such as nonsteroidal anti-inflammatory, anticonvulsant, and antidepressives, associated with removal of local causes of pain when it was necessary. Pharmacological choice was based on the medical, social, and emotional individual history and followed the WHO’s analgesic step-up approach (21).

One patient was diagnosed with BMS secondary to CRT. As with other chronic neuropathic pain conditions, BMS can be managed by pharmacological or by psychological means or by a combination of both (10,29). In this case, we proposed a topical capsaicin and systemic anticonvulsant, but the patient abandoned treatment, similarly to other three cases.

We decided to report four unconcluded cases to discuss the vulnerability of the patients and their families that depend on public health service. A Brazilian study (30) highlighted some issues that affect this vulnerability in the rural population with oral cancer. The knowledge about oral cancer, accessibility to dental care, lifestyle and socioeconomic status are important factors to take into consideration. The majority of patients in our study were from rural or suburb areas, and experienced many of these difficulties, including limited budget to cover the public transportation costs for dental clinic.

According to Epstein *et al.*, OFP in many cases never returns to its baseline status after beginning cancer therapy. These findings were not reproduced in this study where pain scores changed from severe or moderate to no pain in 45,4% of cases. We believe that our approach was successful due to an individual approach and to careful pain diagnosis and treatment.

There are a limited number of studies assessing OFP in HNC patient. Studies addressing the dentist diagnosis and management of pain impact in the overall quality of life of patients with cancer are needed to improve oncological therapy.

## Conclusions

OFP could be associated to tooth, musculoskeletal, and some neurological disorders whose diagnosis and treatment depends on the dentist. In this cases report, oral care approach enabled pain reduction in 86,3% of patients, and pain relief was achieved in 45,4% of them. Oncological patients should be treated by a multidisciplinary team including dentists.
